# Supplementary material for: Identification of photoperiod-induced specific miRNAs in the adrenal glands of Sunite sheep (Ovis aries)
Source: Front Vet Sci. 2022 Jul 22;9:888207. doi: 10.3389/fvets.2022.888207 (PMC9354845; doi:10.3389/fvets.2022.888207)
Supplement: Supplementary file 1 [file Data_Sheet_1.ZIP › Supplementary file/miRDeep2 file.zip!/miRDeep2 file/Know_Structure/SPLP42A3/oar-mir-3959.pdf]

miRBase precursor : oar-mir-3959
Total read count : 13223
oar-miR-3959-5p read count : 11642
oar-miR-3959-3p read count : 1581
remaining reads : 0

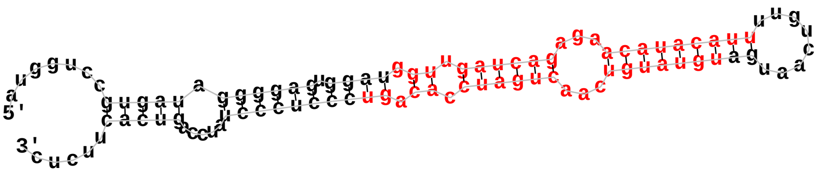

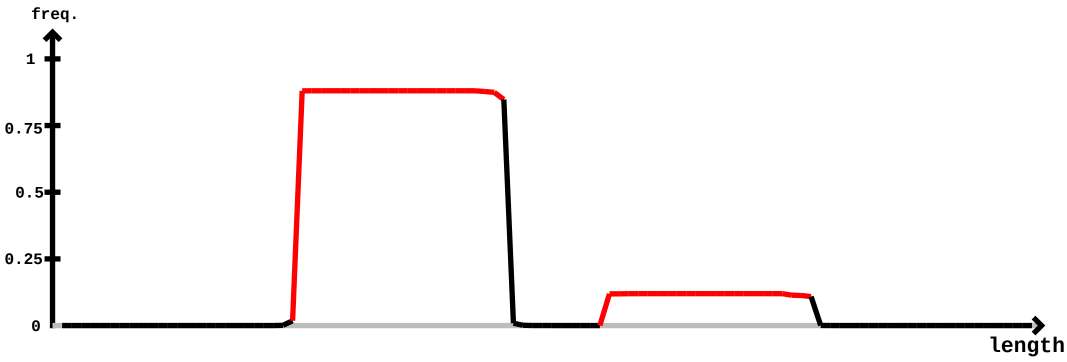

oar-miR-3959-5p

oar-miR-3959-3p

```
5'- augguccgugauaggggaguggauggguugaucagagaacauacauuuugucaaugauguaugucaacugauccacagucccucccuauccagucacuucuc -3'   exp
    .......(((((.((((((.((((.((.((((((...(((((((((........)))))))))...)))))).)).))))))))))).....))))).....   reads   mm   sample
    ......................augguugaucagagaacauac..........................................................   4       0    seq
    ......................augguugaucagagaacauaca.........................................................   6       0    seq
    .......................ugguugaucagagaacau............................................................   1       0    seq
    .......................ugguugaucagagaacauaca.........................................................   3       0    seq
    .......................ugguugaucagagaacauacau........................................................   11      0    seq
    .......................ugguugaucagagaacauacauu.......................................................   213     0    seq
    ........................gguugaucagagaacaua...........................................................   1       0    seq
    ........................gguugaucagagaacauac..........................................................   30      0    seq
    ........................gguugaucagagaacauaca.........................................................   34      0    seq
    ........................gguugaucagagaacauacau........................................................   343     0    seq
    ........................gguugaucagagaacauacauu.......................................................   10877   0    seq
    ........................gguugaucagagaacauacauuu......................................................   103     0    seq
    ........................gguugaucagagaacauacauuuu.....................................................   10      0    seq
    ........................gguugaucagagaacauacauuuuguc..................................................   1       0    seq
    .........................guugaucagagaacauacauu.......................................................   1       0    seq
    .........................guugaucagagaacauacauuuu.....................................................   2       0    seq
    ..........................uugaucagagaacauacauuuugu...................................................   1       0    seq
    ...........................ugaucagagaacauacauuuugu...................................................   1       0    seq
    ........................................................uguaugucaacugauccac..........................   75      0    seq
    ........................................................uguaugucaacugauccaca.........................   16      0    seq
    ........................................................uguaugucaacugauccacag........................   54      0    seq
    ........................................................uguaugucaacugauccacagu.......................   1425    0    seq
    ........................................................uguaugucaacugauccacaguc......................   2       0    seq
    ..........................................................uaugucaacugauccacagu.......................   5       0    seq
    ..........................................................uaugucaacugauccacaguc......................   1       0    seq
    ..........................................................uaugucaacugauccacagucc.....................   2       0    seq
    ...........................................................augAcaacugauccacagu.......................   1       1    seq
```
